# Cross-cultural adaptation and psychometric properties of the Mexican version of the Early Childhood Oral Health Impact Scale (ECOHIS)

**DOI:** 10.1186/s12955-021-01747-3

**Published:** 2021-03-21

**Authors:** Alba Lilia Brambila Montoya, Jessica Klöckner Knorst, Isaac Murisi Pedroza Uribe, Rubén Alberto Bayardo González, Thiago Machado Ardenghi, Carmen Celina Alonso Sánchez

**Affiliations:** 1 Los Altos University Center, Tepatitlán de Morelos, Jalisco México; 2 Department of Stomatology, Faculty of Dentistry, School of Dentistry, Universidade Federal de Santa Maria, Av. Roraima, 1000, Cidade Universitária - 26F, Santa Maria, RS 97015-372 Brazil

**Keywords:** Child, Oral health, Psychometrics, Quality of life, Questionnaire

## Abstract

**Background:**

Instruments adapted for the Mexican population to assess oral health-related quality of life (OHRQoL) in preschoolers remain lacking. This study aimed to cross-culturally adapt and evaluate the psychometric properties of the Mexican version of the Early Childhood Oral Health Impact Scale (M-ECOHIS).

**Methods:**

This cross-sectional study was conducted with preschool children from southern Mexico. The investigation was divided into a transcultural adaptation phase and a validation phase. The M-ECOHIS was completed by the children’s guardians, and clinical data were also evaluated. Reliability was evaluated using tests of internal consistency and test–retest measures, while construct validity was assessed through Spearman’s correlation coefficient between M-ECOHIS scores and self-reported oral health, and through confirmatory factor analysis (CFA). Construct validity was also evaluated through discriminant validity of the M-ECOHIS, which was determined according to questionnaire scores on oral health measures (e.g., dental caries).

**Results:**

A total of 303 preschool children participated in this study. Regarding internal consistency, Cronbach’s alpha was > 0.78 for the child section, family section, and general M-ECOHIS. The general intraclass correlation coefficient (ICC) for test–retest reliability was 0.95. The correlation between the scores obtained on the child and family impact sections was significant with the self-reported oral health status rating. In relation to CFA, all items of the M-ECOHIS confirmed the latent variables. Further, M-ECOHIS scores were associated with the presence of untreated dental caries, indicating that the questionnaire has good discriminant validity.

**Conclusion:**

Our findings suggest that the M-ECOHIS is a valid and reliable instrument for assessing the impact of oral health on quality of life in Mexican preschool children.

## Background

Oral health has been associated with physical, psychological, and social factors [1]. However, despite public health efforts and initiatives to improve oral health globally, oral health problems persist [2], such as dental caries, which remain highly prevalent worldwide and in all age groups [3]. In children aged between 2 and 10 years in Mexico, the average decayed, missing and filling teeth (DMFT) index was 3.8 [4]. Thus, oral health problems continue to pose a significant public health challenge in Mexico.

In this context, the different clinical outcomes are strong predictors of the prevalence of negative impacts on OHRQoL [5–7]. In addition, contextual and socioeconomic factors have been associated with OHRQoL; individuals in the lowest strata of income, housing, and social capital have the poorest OHRQoL [5–7]. OHRQoL has been defined as a multidimensional construct that reflects the extent of the impact that health or oral disease has on the daily life and well-being of individuals [8, 9]. Thus, OHRQoL is considered an essential parameter in the evaluation of oral health, since its multidimensionality has been increasingly singled out as an integral factor of oral health [1, 9].

Several instruments—most being questionnaires—have been developed to measure OHRQoL, which is also called dental indicators [10]. However, most questionnaires measuring OHRQoL have been developed for adult populations. Nevertheless, an interest grew in the impact of oral diseases on children’s quality of life, especially given that assessment measures differ between children and adults [11]. Thus, instruments were developed and validated for children, including those younger than 5 years old [12].

Some such instruments whose validity and reliability has been confirmed are the Michigan COHRQoL scale, the Early Childhood Oral Health Impact Scale (ECOHIS), the Scale of Oral Health Outcomes for 5-year-old children (SOHO-5), and the Pediatric Oral Health-Related Quality of Life (POQL), as reported in a recent review that presented a practical guide to measurement tools of OHRQoL in preschool children [13]. Among the tools available, the ECOHIS is considered robust, since it was designed and tested for use at the population level and is applicable in a variety of situations [13]. In addition, another study made a standardized comparison among tools for preschoolers and concluded that the ECOHIS had the highest overall quality score in terms of conceptual model, reliability, and validity [14]. For these reasons, the ECOHIS is a good instrument for measuring OHRQoL in preschoolers.

The Early Childhood Oral Health Impact Scale (ECOHIS) was developed for use in epidemiological studies and validated in the U.S. It assesses the impact of oral health conditions and past experiences with dental treatments on the quality of life of preschool-aged children and their parents or other family members [15]. The questionnaire consists of 13 questions divided into two sections: the impact of oral health on the child (child impact scale [CIS]; 9 questions) and the impact on the family (family impact scale [FIS]; 4 questions). The scale has Likert-style response options to record the frequency of a given event in the child’s life: 0 = never; 1 = rarely; 2 = occasionally; 3 = often; 4 = very often; 5 = don’t know. CIS and FIS ECOHIS scores ranged from 0–36 and 0–16, respectively; higher scores indicate a poorer OHRQoL.

In recent years, several versions of the ECOHIS have been translated, adapted, and validated for different languages [16, 17]. Among them is a version adapted for Spanish populations for administration in Latin America, allowing for a reliable and valid application in Latin American countries [18]. However, to our knowledge, no version to date has been adapted and validated specifically for the Mexican population. Thus, the present study aimed to cross-cultural adapt and evaluate the psychometric properties of the ECOHIS for applicability in Mexican preschool children. We hypothesized that the Mexican version of the ECOHIS has good psychometric properties.

## Methods

### Ethical considerations

This research is in accordance with the Declaration of Helsinki and with NOM-012-SSA3-2012, which establishes the criteria for the ethical execution of health research projects in humans. This project was also presented to the ethics committee of the Centro Universitário de Los Altos. All parents or guardians signed an informed consent form.

### Study design and sample

Data for this cross-sectional study were collected in 2017 from a sample of 303 preschool children aged 3–5 years in the city of Tepatitlán de Morelos, which is located in the southern high region of Jalisco, Mexico. According to the 2015 National Population Census conducted by the Institute of Statistical and Geographic Information of Jalisco (IIEG), the city’s population is 141,322. Ten of the Tepatitlán de Morelos’ 55 preschools, six of which are public and four of which are private, were randomly selected. The eligibility criteria were that parents or guardians understood Spanish and did not have any cognitive or other relevant limitations.

The sample size requirements were evaluated according to the power calculation for this study’s sample. The power calculation accounted for an alpha error probability of 0.05 and overall ECOHIS scores of 1.8 (standard deviation [SD] 3.7) in the non-exposed group (without untreated dental caries) and 4.7 (SD 5.5) in the exposed group (with untreated dental caries), resulting in a sample power of 99.9%. We also calculated the minimum sample required for the expected value of ICC using the PASS software (PASS 2020, NCSS). Considering a significance level of 0.05, a power of at least 0.8, and a minimum detectable ICC of 0.2, a sample of 152 subjects is required when the agreement in the null hypothesis is pre-specified as equivalent to 0. For the validation phase, the inclusion criteria were preschool children of either sex with good general health status and complete temporary dentition, informed consent from parents or guardians, and completion of a socioeconomic data questionnaire by the parent or guardian.

### Preliminary phase: transcultural adaptation of ECOHIS

The ECOHIS was originally developed in English and validated in North Carolina [15]. It evaluates the impact of oral health conditions and experiences with dental treatments on the quality of life of preschool children (3–5 years old) and their parents or other family members. The ECOHIS was translated into Spanish for use in Latin America, resulting in a reliable and valid application [18].

A committee comprising four pediatric dentists with expertise on health-related quality of life instruments convened to discuss the semantics of the Spanish-translated version of the ECOHIS, as shown in previous studies [16, 18]. Some word substitutions were made for ease of understanding: a) “occasionally” for “sometimes”; b) “often” for “frequently”; c) “very often” for “commonly”; d) “difficulties” for “difficulty”; e) “by” for “due to”; f) “he has missed days of attendance” for “he has been absent”; g) “he has demonstrated” for “he has been”; h) “the smile” for “smile”; i) “it has been disturbed” for “it has been disturbed/worried”; j) “it has had to occupy “ for “it has occupied”; k) “they have determined economic impact on their family” for “they have affected the economy on their family/home”.

The resultant adapted version was pilot-tested in a convenience sample of 30 parents of pediatric patients who attended the clinic of the Pediatric Dentistry of the Centro de Atención Médica Integral de Centro Universitario de los Altos de la Universidad de Guadalajara. The test was reapplied to the same participants one week after the first application in order to compare and contrast the results.

### M-ECOHIS and socioeconomic questionnaire

The semantically adjusted questionnaire and the socioeconomic questionnaire were administered to the 303 parents or guardians who did not participate in the preliminary phase. The M-ECOHIS and the socioeconomic questionnaire were answered by one of the parents or guardians of the child via in-person or telephone interviews conducted by trained interviewers. A previous study showed that the ECOHIS was valid when administered via telephone, with no significant differences relative to in-person administration [19]. The questionnaires were completed prior to the child’s oral clinical examination. To evaluate test–retest reliability, the M-ECOHIS was completed on two separate occasions (seven days apart) by 30 caregivers, similar to the intervals used in previous studies [12, 16, 17].

Demographic and socioeconomic variables included sex (male or female), age (years), type of school (public or private), maternal educational attainment, and household income. Maternal education was categorized into either < 8 or ≥ 8 years of formal education. Household income was evaluated in Mexican peso (Mex$26.60 was approximately equivalent to USD$1.00) and dichotomized by the median for the analysis.

### Oral clinical examination

Clinical data were collected from dental examination records by four researchers who were previously calibrated for the assessment of dental caries according to the World Health Organization (WHO) criteria [20] by theoretical class, in vitro practice with natural teeth, and completion of clinical examinations on five children in the pediatric dentistry clinic. Clinical examinations were performed in the preschool, with the aid of a flashlight, a tongue depressor, gauze, and periodontal probes (“ball point”). Inter- and intra-examiner reproducibility for clinical variables was considered good (Kappa > 0.7).

The presence of dental caries was evaluated according to the number of decayed, missing, and filled teeth (dmft index). For the analysis, the dmft ≥ 1 and also the presence of untreated dental caries (recorded as a non-zero d component in the dmft index) were considered. We also assessed the presence of dental trauma in the upper incisors and of occlusal disorder (lip coverage and anterior open bite) [20].

### Statistical analysis

Data were analyzed with Stata 14 (StataCorp. 2014. Stata Statistical Software: Release 14.1. College Station, TX: StataCorp LP). Demographic, socioeconomic, and clinical oral health characteristics were described using Stata’s “svy” command for complex data samples.

### Validity and internal consistency reliability assessment

Test–retest reliability was evaluated through tests of internal consistency. The internal consistency of the CIS and FIS and overall scores was assessed using Cronbach's alpha, with values ≥ 0.70 deemed acceptable for comparisons between groups [21]. Test–retest reliability was determined through the calculation of the Intraclass Correlation Coefficient (ICC) for the scores on the CIS and FIS, as well as overall. ICC values ≥ 0.60 were deemed good, and those ≥ 0.80 were deemed excellent [22].

### Construct validity

Construct validity was assessed through convergent validity, confirmatory factor analysis (CFA), and discriminant validity [23]. The convergent validity of M-ECOHIS was assessed through Spearman’s correlation coefficient between CIS and FIS M-ECOHIS scores and self-reported oral health. The correlation with the overall M-ECOHIS scores was also assessed. In addition, CFA was performed to evaluate the measurement model and the relationships among the scale’s 13 items and the latent variables for the CIS and FIS. For this approach, maximum likelihood (ML) estimation was used. Modification indices (MI) were used for statistical fit, as well as to evaluate correlations between items. The standardized coefficient (SC) is represented by beta weights, indicating a value of 0.10 as small, of 0.30 as medium, and of > 0.50 as high factorial load [24]. The goodness‐of‐fit was measured using root mean square error of approximation (RMSEA), the comparative fit index (CFI), and the Tucker‐Lewis index (TLI). A RMSEA value < 0.05 and a CFI and TLI value < 0.90 denotes adequate fit. The standardized root mean square residual (SRMR) indicates an adequate fit when a value falls below 1.0 [24].

Construct validity was also assessed through the discriminant validity of the M-ECOHIS, determined according to questionnaire scores with oral health measures (dental caries, dental trauma, lip coverage, and anterior open bite). We hypothesized that children with higher levels of oral disease were more likely to have higher scores in the CIS, FIS, as well as in the overall M-ECOHIS. Adjusted Poisson regression models were used to test these hypotheses. Variables that presented *p* < 0.20 in the unadjusted analyses were considered to be adjusted to the model (household income, maternal education, and sex). The results are presented as a rate ratio (RR) with 95% confidence interval (95% CI). Variables with *p* < 0.05 were considered statistically significant.

## Results

A total of 303 preschool children participated in this study. Table 1 displays characteristics of the sample according to demographic, socioeconomic, and clinical variables. The sample comprised a similar distribution of males and females, with the majority being > 5 years of age (53%) and being enrolled in a public school (56.4%). Regarding maternal educational attainment, most mothers had > 8 years of formal education. Approximately 55% of the children had untreated dental caries, and 11.3% had an open anterior bite.

**Table 1:** Characteristics of the sample according to demographic, socioeconomic, and oral health variables (n = 303)

Variables	n	(%)*
*Demographic and socioeconomic variables*		
Sex		
Male	155	(51.0)
Female	148	(49.0)
Age (years)		
3	22	(8.7)
4	118	(38.3)
5	163	(53.0)
Type of school		
Public	179	(56.4)
Private	124	(43.6)
Maternal education		
< 8 years of formal education	43	(14.2)
> 8 years of formal education	260	(85.8)
Household income in Mex$^a^		
< 7000	119	(50.1)
> 7000	111	(49.9)
*Clinical variables*		
Dental caries experience		
Dmft = 0	166	(55.0)
Dmft ≥ 1	137	(45.0)
Untreated dental caries		
Absence	159	(52.1)
Presence	144	(47.9)
Traumatic dental injury		
Absence	295	(97.5)
Presence	8	(2.5)
Lip coverage		
Adequate	275	(90.8)
Inadequate	28	(9.2)
Anterior open bite		
Absence	270	(88.7)
Presence	33	(11.3)

*Taking into account the sample weight; Values below 303 due to missing data

^a^Mex$, Mexican peso (Mex$26.60 was approximately equivalent to USD$1.00)

The descriptive distribution of responses to the M-ECOHIS is presented in Table 2. Regarding the CIS, “pain in the teeth, mouth, or jaws” (14.4%) was the most frequently reported impact, followed by “had difficulty pronouncing any words” (7.9%). Regarding the FIS, “a financial impact on your family” (12.6%) was the most frequently reported impact. The impact that yielded the most “never” responses was that of “missed preschool, daycare, or school” (94.1%), followed by “avoided talking” (91.3%). The overall mean M-ECOHIS score was 3.2 (standard error [SE] 0.23). The overall scores were 1.93 (SE: 0.18) and 1.28 (SE: 0.08) for the CIS and FIS, respectively.

**Table 2:** Descriptive distribution of responses to the M-ECOHIS (n = 303)

Impact	Never	Rarely	Occasionally	Often	Very often	Mean (SE)
	N (%)	N (%)	N (%)	N (%)	N (%)	
Child section						
How often has your child had pain in the teeth, mouth, or jaws?	197 (65.0)	62 (20.5)	37 (12.2)	6 (2.0)	1 (0.3)	0.52 (0.03)
*How often has your child […] because of dental problems or dental treatments?*						
Had difficulty drinking hot or cold beverages	251 (82.8)	32 (10.5)	17 (5.6)	3 (1.0)	0 (0.0)	0.24 (0.04)
Had difficulty eating some foods	242 (79.9)	43 (14.2)	13 (4.3)	5 (1.7)	0 (0.0)	0.27 (0.04)
Had difficulty pronouncing any	255 (84.2)	24 (7.9)	18 (5.9)	3 (1.0)	3 (1.0)	0.25 (0.03)
Missed preschool, daycare or school	285 (94.1)	9 (3.0)	8 (2.6)	1 (0.3)	0 (0.0)	0.08 (0.01)
Had trouble sleeping	268 (88.5)	23 (7.5)	10 (3.3)	2 (0.7)	0 (0.0)	0.16 (0.01)
Been irritable or frustrated	269 (88.8)	19 (6.3)	12 (4.0)	2 (0.7)	1 (0.0)	0.18 (0.02)
Avoided smiling or laughing	276 (91.1)	16 (5.3)	9 (3.0)	2 (0.7)	0 (0.0)	0.12 (0.02)
voided talking	284 (93.7)	15 (5.0)	3 (1.0)	1 (0.3)	0 (0.0)	0.08 (0.2)
Family section						
*How often have you or another family member […] because of your child’s dental problems or treatments?*						
Been upset	251 (82.4)	29 (9.6)	19 (6.3)	3 (1.0)	1 (0.3)	0.26 (0.01)
Felt guilty	247 (81.5)	27 (8.9)	19 (6.3)	6 (1.2)	4 (1.3)	0.32 (0.04)
Taken time off from work	239 (78.8)	34 (11.2)	26 (8.6)	2 (0.7)	2 (0.7)	0.32 (0.04)
How often has your child had dental problems or dental treatments that had a financial impact on your family?	236 (77.8)	29 (9.6)	31 (10.2)	5 (1.7)	2 (0.7)	0.36 (0.02)

*SE* standard error

**Fig. 1:**
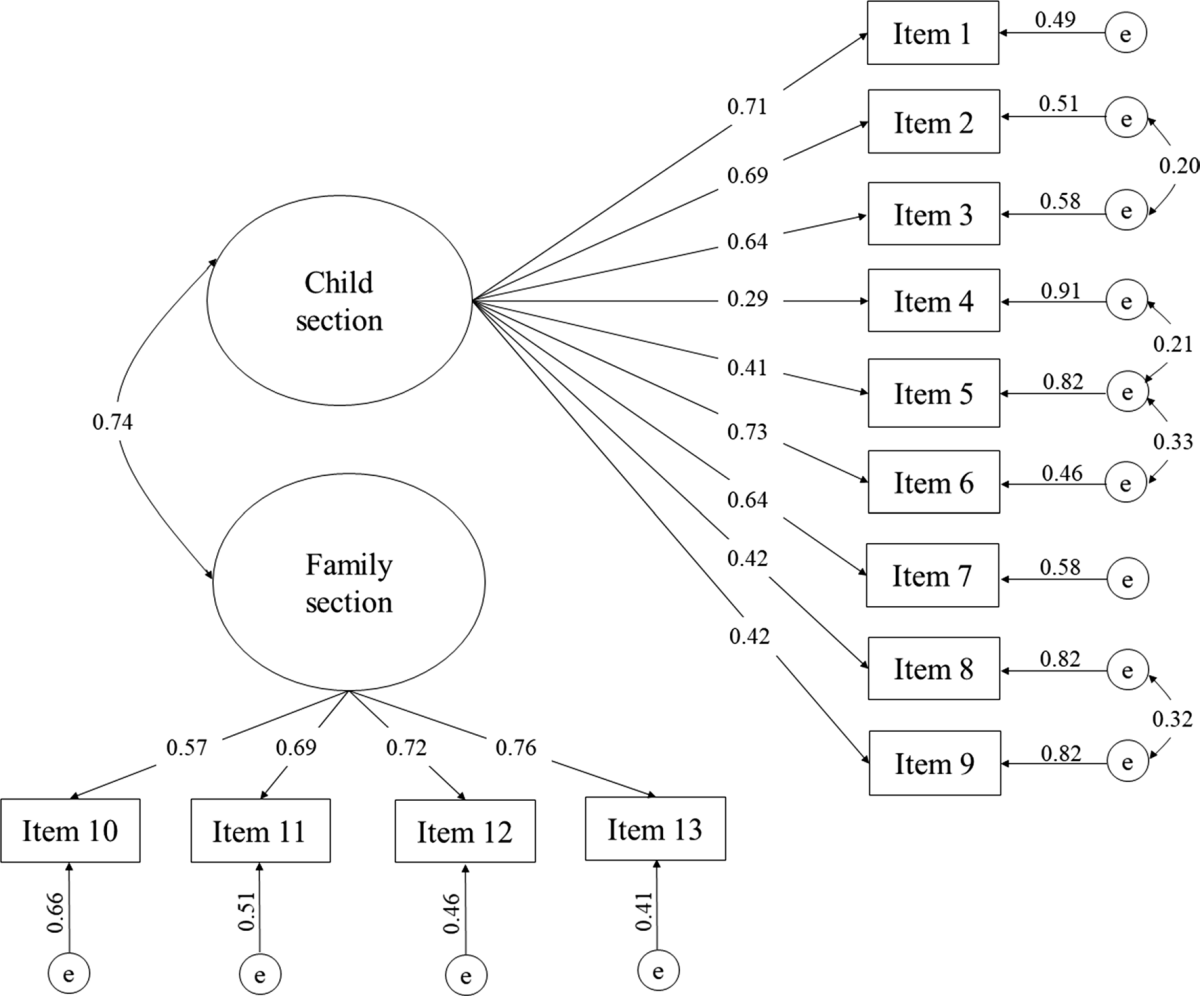
Confirmatory factor analysis (CFA) of the M-ECOHIS

Table 3 presents findings for internal consistency, reproducibility, and convergent validity of the M-ECOHIS. Regarding internal consistency, Cronbach’s alpha was 0.80 for the CIS, 0.78 for the FIS, and 0.85 for the overall M-ECOHIS. The general ICC for the test–retest reliability (reproducibility) was 0.95. The ICC was 0.60 and 0.90 for the CIS and FIS, respectively. Spearman's correlation between the M-ECOHIS and the child’s self-reported oral health status showed a significant convergent validity when related to overall M-ECOHIS scores and to the CIS and FIS (*p* < 0.05). In relation to CFA (Fig. 1), all items of the M-ECOHIS confirmed the latent variables in the child and family impact section (*p* < 0.01). The majority of the SCs presented high values (> 0.5), confirming good construct validity. The global adjustments of the parsimonious model were: SRMR = 0.05, CFI = 0.90, TLI = 0.87, and RMSEA = 0.08 (0.07–0.09).

**Table 3:** Findings for internal consistency, reproducibility, and convergent validity of the M-ECOHIS

Factors	Child section	Family section	Overall
Cronbach’s alpha coefficient	0.80	0.78	0.85
Intra-class correlation coefficient	0.60	0.90	0.95
Spearman’s correlation coefficient^a^	0.42*	0.34*	0.43*

^a^Convergent validity with oral health status rating; *p < 0.05

Findings for the discriminant validity of the M- ECOHIS are shown in Table 4. The results indicate that children who have experienced caries (dmft > 1) had higher scores on the M-ECOHIS as a whole (RR 3.46; 95% CI 2.45–4.88), as well as in the CIS (RR 3.68; 95% CI 1.84–8.46) and FIS (RR 3.58; 95% CI 2.24–5.71). In addition, children who presented with untreated dental caries had the highest scores on the M-ECOHIS (RR 2.41; 95% CI 1.59–3.64). These results indicate good discriminant validity of the M-ECOHIS. The other clinical variables were not associated with M-ECOHIS scores.

**Table 4:** Findings for the discriminant validity of the M-ECOHIS (n = 303)

Variables	Child section		Family section		Overall	
	Mean (SE)^a^	RR^b^ (95% CI)^c^	Mean (SE)	RR (95% CI)	Mean (SE)	RR (95% CI)
Dental caries experience						
Dmft = 0	0.92 (0.11)	1.00	0.62 (0.08)	1.00	1.54 (0.18)	1.00
Dmft ≥ 1	3.18 (0.22)	3.46 (2.45–4.88)*	2.08 (0.25)	3.68 (1.84–8.46)*	3.26 (0.39)	3.58 (2.24–5.71)*
Untreated dental caries						
Absence	1.11 (0.09)	1.00	0.72 (0.06)	1.00	1.83 (0.13)	1.00
Presence	2.83 (0.25)	2.24 (1.30–3.87)*	1.89 (0.15)	2.71 (1.83–4.03)*	4.73 (0.30)	2.41 (1.59–3.64)*
Traumatic dental injury						
Absence	1.89 (0.18)	1.00	1.26 (0.08)	1.00	3.1 (0.24)	1.00
Presence	3.5 (0.96)	1.21 (0.84–1.74)	1.90 (1.00)	1.34 (0.57–3.13)	5.4 (1.95)	1.26 (0.73–2.17)
Lip coverage						
Adequate	1.85 (0.18)	1.00	1.28 (0.10)	1.00	3.1 (0.25)	1.00
Inadequate	2.85 (0.25)	1.45 (0.82–2.58)	1.27 (0.16)	0.94 (0.48–1.84)	4.0 (0.23)	1.25 (0.77–2.01)
Anterior open bite						
Normal	1.8 (0.20)	1.00	1.30 (0.09)	1.00	3.1 (0.26)	1.00
Accentuated	2.3 (0.19)	1.11 (0.52–2.36)	1.22 (0.10)	0.78 (0.57–1.07)	3.5 (0.27)	0.98 (0.56–1.72)

Taking into account the sample weight

^a^*SE* standard error

^b^*RR* rate ratio determined using Poisson regression model (adjusted by household income, maternal education, and sex)

^c^*CI* confidence interval

*p < 0.05

## Discussion

This study aimed to validate a Mexican version of the ECOHIS questionnaire in a sample of Mexican children and their caregivers. As the main result, M-ECOHIS demonstrated good consistency, reproducibility, and construct validity, in accordance with our hypotheses. Although the ECOHIS has been validated in several countries, instruments validated for Mexican populations to assess OHRQoL in preschoolers were lacking.

Approximately half of the children in our sample had untreated dental caries, which may have biased the finding that “pain in the teeth, mouth, or jaw” was the impact most frequently reported in the CIS. Previous studies indicate that children with untreated dental caries may have difficulty eating, sleeping, and socializing, in addition to affecting self-confidence, weight, and growth, thereby degrading quality of life [25–27]. In the FIS, the item “they affected the economy in your family/home” was the most frequent impact reported, which could suggest that oral health can substantially affect children’s quality of life and the household’s finances. These findings corroborate those of previous studies [12, 17], indicating that the M-ECOHIS can be compared with other versions of the ECOHIS.

Test–retest reliability (reproducibility) was adequate, with an ICC of 0.95, similar to the value reported in Peru [12], Brazil [28], and Turkey [29], and higher than the values reported in the U.S. [15], China [30], and Iran [25]. In this context, the Mexican version of the ECOHIS showed excellent reproducibility, as it is capable of producing consistent results when administered to the same person at two different times [31]. Regarding internal consistency, Cronbach's alpha was 0.80 for the CIS, 0.78 for the FIS, and 0.85 for the overall M-ECOHIS, which indicates good internal consistency (0.70) [24]. Other ECOHIS validation studies have reported similar values [16, 25, 28, 30].

With respect to convergent validity, Spearman's correlation coefficient between the M-ECOHIS and the child’s self-reported oral health status showed significant convergent validity, but weak (< 0.49) [23]. However, the results are similar to those of the original study of the development of the ECOHIS [15], as well as to those of studies performed in Brazil [16] and Latin American communities [18]. Furthermore, all M-ECOHIS items confirm the latent variables in the impact of the CIS and FIS, thereby indicating good construct validity [24].

In addition, our findings demonstrated that children with a history of caries or with untreated caries had significantly higher overall scores on the M-ECOHIS, supporting the questionnaire’s discriminant validity. Similar results have been observed in other versions [12, 27, 29, 30]. As described, individuals with dental caries are more likely to have dental pain and difficulties sleeping and eating, which can directly worsen OHRQoL [32]. The other clinical variables were not associated with the M-ECOHIS scores. However, previous validation studies have only relied on dental caries for evaluating the discriminant validity of the questionnaire [12, 15, 16], in accordance with our findings.

The present study was carried out in a sample of children aged 3–5 years, despite the fact that the instrument was developed and validated for use in children aged 0–5 years [13], which can limit the generalizability of our findings. However, obtaining cooperation from children younger than 3 years of age can prove challenging, and only a minority in this age group attend nursery school. Another limitation is the relatively brief interval between the first and second application of the questionnaire. Nevertheless, no consensus has been reached on the most appropriate period for assessing the reliability of patient-reported outcome (PRO) instruments [33]. Furthermore, The 7-day interval used in our study was also used in previous validation studies [12, 16, 17]. Our study, nonetheless, had strengths; the application of this instrument in preschool children renders possible the evaluation of the effectiveness of oral health programs, in addition to the prioritization of investments, the evaluation of treatment results, and comparisons of oral health throughout childhood [17].

Future studies in Mexican preschool children are encouraged to apply this recently validated questionnaire for evaluation of OHRQoL. The application of the M-ECOHIS scale can be useful for clinical and epidemiological research for exploring more comprehensively which factors impact on subjective measurements of preschool children in this population.

## Conclusion

The M-ECOHIS version was cross-culturally adapted into Spanish by modifying some terminology and syntax. Our findings suggest that the M-ECOHIS is a valid and reliable instrument for assessing the impact of oral health on quality of life in preschool children aged between 3 and 5 years. Ultimately, the M-ECOHIS can be a useful tool for future studies on oral health in Mexican pediatric populations.

## Abbreviations

ECOHIS: Early Childhood Oral Health Impact Scale
CFA: Confirmatory factor analysis
OHRQoL: Oral Health-related Quality of Life
WHO: World Health Organization
DMFT: Decayed, missing and filling teeth
SD: Standard deviation
SE: Standard error
ICC: Intraclass correlation coefficient
ML: Maximum likelihood
MI: Modification indices
SC: Standardized coefficient
RMSEA: Root mean square error of approximation
CFI: Comparative Fit Index
TLI: Tucker–Lewis Index
SRMR: Standardized root mean square residual
RR: Rate ratio
